# The ‘telegraphic schizophrenic manner’: Psychosis and a (non)sense of time

**DOI:** 10.1177/0961463X20916109

**Published:** 2020-05-07

**Authors:** Michael J Flexer

**Affiliations:** Wellcome Centre for Cultures and Environments of Health, Queen’s Building, University of Exeter, Exeter, UK

**Keywords:** Psychosis, semiotics, deixis, Tralfamadorean time, Deleuzean Event, autonoesis and mental time travel, episodic memory, Marge Piercy, Kurt Vonnegut

## Abstract

This paper reads Vonnegut’s *Slaughterhouse-Five* and Piercy’s *Woman on the Edge of Time* as stories of deictic temporal crises. It critically examines the texts, exploring their representations of mental time travel (MTT), and places them into dialectic with health sciences research on autonoesis and episodic memory deficits in people with lived experience of mental health disorders, particularly psychosis or ‘schizophrenia’. The paper uses this dialectic to interrogate how atypical MTT is diagnostically and clinically rendered as pathological, and indicative of psychosis in particular. Similarly, it mines these fictional representations for the insights they might provide in attempting to understand the phenomenological reality of temporal disruptions for people with lived experience of psychosis. The paper moves on to incorporate first-person accounts from people with lived experience, and uses these to refine a Deleuzean static synthesis of time constructed around the traumatic Event and the Dedekind ‘cut’. The paper concludes with some suggestions as to how the literary texts offer possible insights into the experience of people living with ‘psychotic’ temporal disruptions, and in particular how to re-invest their deictic relations to establish functioning fixity and stability of the self in time.

Billy is spastic in time, has no control over where he is going next, and the trips aren’t necessarily fun. He is in a constant state of stage fright, he says, because he never knows what part of his life he is going to have to act in next. (Vonnegut, 1991: 17)

The first time. Was there a once? The dreams surely began with an original; yet she had the sense, the first morning she awakened remembering, that there were more she had not remembered, a sensation of return, blurred but convincing. (Piercy, 1979: 33)

## The loss of the once; going ‘spastic’ in time

Listen: *Slaughterhouse-Five* Kurt Vonnegut’s 1969 self-described ‘anti-war book’ (p.3) doesn’t begin, or rather begins at least three times, and perhaps is always beginning. As suggested by Vonnegut’s use of the Yon Yonson playground rhyme recursively progressing ‘to infinity’ (p.2) as a metaphor for his writing process, always beginning is also a form of never beginning and of never ending. Momentarily leaving aside the many temporal spasms within the diegetic^1^ sequence of events in *Slaughterhouse-Five*, the text as extra-diegetic artefact offers up three different moments of beginning: the paratextual frontispiece (a self-consciously anachronistic device); the opening chapter on Vonnegut’s attempts at starting and finishing writing his book about Dresden; the second chapter that begins protagonist Billy Pilgrim’s tale of time travel and alien abduction with its instant interpolating cry of ‘listen’ (p.17). This third beginning, and the start of the narrative of Billy himself, abruptly situates the reader and the narrative voice in a shared here and now. The very suddenness indicates an inherent anxiety about that shared here and now, and points to a deep, implicit inverse relationship between temporal displacement (being ‘unstuck in time’) and what this paper will call deictic fixity.

Deixis is a useful way of tracking how the effect of being ‘unstuck in time’ is produced in and experienced by subjects, whether they are Billy, the narrative voice, the reader or people out in the world. In semiotic terms, deixis concerns meaning-generation dependent on the temporal, spatial and interpersonal location and validation of an addresser; from a social semiotic perspective, deixis further encompasses the range of socio-cultural connections, conventions and complicities necessary for ‘I am here now’ to be a meaningful statement. To be recognised in a deictic relationship with an interlocutor is to be constituted as a self in a time that extends beyond the self. To not be recognised, or to be effaced, and denied status in deixis, is to become unstuck.

As with *Slaughterhouse-Five*, Marge Piercy’s 1976 speculative fiction (SF) *Woman on the Edge of Time* begins and then unbegins and rebegins with an analeptic second chapter, a flashback in time that recasts the events of the first chapter and restages the cycles of re-incarceration inevitable for a working-class woman of colour with a mental health diagnosis in 1970s America. The return to incarceration for protagonist Connie, like the returning trauma of losing her daughter or the deaths of her lovers, drains ‘the once’ of all substance and presence. Without any ‘once’, no new narrative can be forged, and it is a rediscovery of a ‘once’ and the possibility of different futures – the survival or destruction of the utopian Mattaposett community – that gives the novel its dramatic stakes and structure. Locating and experiencing a ‘once’ is essential to having any kind of self that can be located temporally between a receding past and an approaching future; vital for ‘trans-temporal sameness,’ to use the language of cognitive neuroscience (Metzinger, 2007). Denied the ‘once’, Piercy’s protagonist Connie is excluded to the very ‘edge of time’. Re-establishing some kind of preliminary temporal and existential firstness is a necessary precondition of any agency and of any future opening up for Connie. When she first reconnects with Luciente – her time-travelling visitor from a possible future – Connie emphasises this new firstness: ‘This is the first time I’ve been by myself since the first night’ (p.63).

Incarceration in Piercy’s text enacts a crisis of deixis perhaps similar to that presupposed by Vonnegut’s text:

She tried to tell the nurse who gave her the injection, the attendants who tied her to the stretcher, that she was innocent, that she had a broken rib, that Geraldo had beaten her. It was as if she spoke another language, that language Claud’s buddy had been learning that nobody else knew: Yoruba. They acted as if they couldn’t hear you. If you complained, they took it as a sign of sickness. “The authority of the physician is undermined if the patient presumes to make a diagnostic statement.” She had heard a doctor say that to a resident, teaching him not to listen to patients. (p.19)

Connie – as a dialogic subjective self – is effaced. Even her body is overwritten. Without having her locutionary participation, she cannot translate her phenomenological symptom (a broken rib) into a clinically recognised sign indicating the need for treatment. Refusing deictic relations results in exclusion from or mis-reading within the medical semiotic (Foucault, 2003: 110–111). It is specifically language, according to Roland Barthes, that allows for the reification of subjective patient symptom into medical sign (1994: 205). In both *Woman on the Edge of Time* and *Slaughterhouse-Five*, therefore, a ‘brief urgent message’ is going unheard (Vonnegut, 1991: 64) and refusal to receive the communication, erasure of the interlocutor’s agency and temporal disruption are profoundly intertwined.

**Figure 1.**
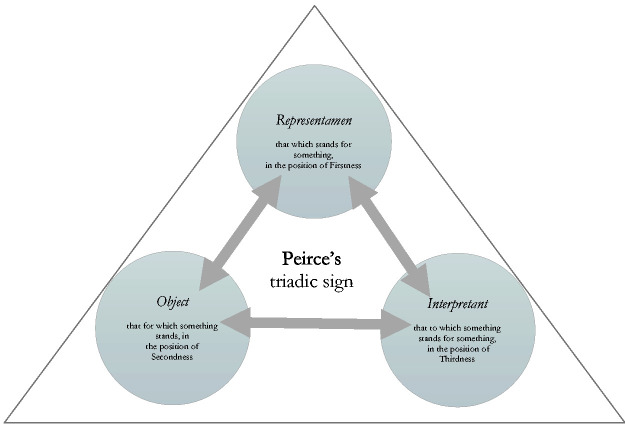
Peirce’s triadic sign.

**Figure 2.**
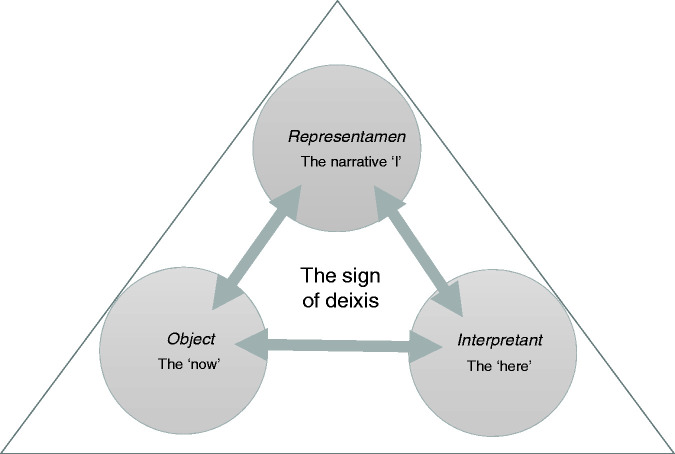
The sign of deixis.

The triadic relationship of deixis, fusing a narrative voice with a ‘here and now’, can be modelled according to the triadic sign structure proposed by semiotician Charles Peirce, whereby a sign is ‘something that stands to somebody for something in some respect or capacity’ (1955: 99). The three components of the sign – *Representamen*, *Object* and *Interpretant* – are combined in an indivisible unity as shown in Figure 1. The *Representamen* is that something which stands to somebody (the *Interpretant*, as functional space) for something in some respect or capacity, i.e. the *Object*. In the deictic sign, proposed in Figure 2, there is a mutually dependent, indivisible relationship whereby the voice which speaks stands to listeners in a particular context (a ‘here’, be it textual or material) for a shared, collective temporal moment (a ‘now’). If the *Interpretant* detaches, through an estrangement between the voice and the ‘here’, then the ‘now’ is lost. It is important to understand this ‘here and now’ as a semiotic, textual effect, as Barthes describes it:

[T]emporality is only a structural category of narrative (of discourse), just as in language [*langue*] temporality only exists in the form of a system; from the point of view of narrative, what we call time does not exist, or at least only exists functionally as an element of the semiotic system. (1977: 99)

A temporal crisis is therefore a textual and a semiotic crisis, encapsulated in this deictic sign. With this model in place, this paper can explore the functioning, the characteristics, the symptoms and the implications of the temporal crisis in ‘psychosis’ encapsulated in Vonnegut’s phrases ‘the telegraphic schizophrenic manner’^2^ and ‘spastic in time’.^3^

Taking this model of the crisis in deictic relations as a point of departure, this paper will interrogate these two SF texts, testing their differing but complementary representations of temporal disruptions and dislocations as potentially illustrative of the lived experience of one possible element of psychosis. From here, the paper will examine how these temporal disruptions manifest in the grammatical and deictic features of the speech of people living with a diagnosis of psychosis. These findings will then be used to refine a model of Deleuze’s Kantian-infused three syntheses of time, proposing that these literary texts and the speech of people with lived experience are not pathologically erroneous, but mimetically accurate and clinically valuable representations of the temporal phenomenology possible – but not universal or inevitable – in psychosis. Noting successful therapeutic interventions with magical realist genre texts in supporting people with lived experience of ‘psychosis’, this paper will then conclude with some suggestions as to how these two SFs offer insights into the experience of people living with ‘psychotic’ temporal disruptions, and in particular how to re-invest their deictic relations to establish functioning fixity and stability of the self in time. In theorising about time, drawing on the semiotic, textual analysis, this paper will close with a geometric modelling of time, aiming to accommodate both the boundless, infinite time of pure time and timelessness, and the strict, bounded finite subjective experience of time. The intention here is: to give an introduction to semiotic modes of thinking for an interdisciplinary readership; to open up any insights into the phenomenology of ‘psychosis’ latent in these bestselling SF texts; to frame the ensuing re-appraisal of temporal phenomenology of ‘psychosis’ in a way that is consciously de-pathologised and de-pathologising; and to offer a levelling structure that allows for mutual identification and recognition across the supposedly incommensurable gap or rupture of ‘madness’.

## A pathology of the present/presence

Cheekily, almost promiscuously, *Slaughterhouse-Five* invites diagnoses for its protagonist. The superficial similarities between Billy’s ‘spasticity’ and posttraumatic stress disorder (PTSD) have earned him this diagnostic label repeatedly (Collado-Rodriguez, 2009: 65; Luckhurst, 2014: 161; Tang, 2018: 9). Stepping momentarily away from the (contested) language of psychiatric diagnostic categories, Billy’s temporal displacement and dissociation share obvious similarities with first-person accounts of the trauma of warfare. Billy was a fictional witness to a real act of traumatic violence in World War Two that Vonnegut himself actually experienced: the brutal firebombing of Dresden by British and American air forces. However, Svetlana’s Alexievich’s (2018) *The Unwomanly Face of War* – an oral history of female Soviets in World War Two – abounds with real-life examples that are strikingly similar to Billy’s representation in *Slaughterhouse-Five*. Consider this fractured recollection of being a Soviet soldier under Nazi bombardment, which exhibits the temporal ‘spasticity’ and deictic slippage between dream and reality characteristic of Billy’s way of being in the world and time:

I often have dreams nowadays … I know I have them, but I rarely remember them. But I’m left with the impression that I’ve been somewhere … And come back … In a dream, what took years in real life takes just a second. And sometimes I confuse dream and reality … I think it was in Zimovniki, I just lay down for a couple of hours, when the bombardment began. Eh, you! Fuck it all … Better to be killed than spoil the pleasure of a two-hour nap. There was a big explosion somewhere nearby. The house rocked. But I still went on sleeping … (Alexievich, 2018: 182)

Temporal ‘spasticity’ is inherent to working clinical and research formulations of PTSD, albeit in more conservative and circumspect terms, with dissociative reactions such as ‘flashbacks’ mentioned explicitly under criterion B3 in the current edition of the *DSM* (American Psychiatric Association, 2013: 271). Zlomuzica et al. (2018) conceptualise PTSD as a pathology of mental time travel (MTT) ‘defined as the ability to recollect past events from episodic memory (MTT into the past) and to anticipate or imagine events in the future (MTT into the future)’ (p.43). When the authors argue that a deficit in MTT ability undermines episodic and prospective memory and reduces the practical agency of those so impaired, translating into ‘difficulties in planning and structuring everyday activities and […] compromised social problem-solving’ (Zlomuzica et al., 2018: 50–51), it is easy to repurpose this as a description of Billy in his depressive, distracted, displaced and de-agentive state.

Billy also receives serious head trauma in an airplane accident, of which he is the only survivor; significantly, it is only from this moment onwards – from the perspective of other characters in the text – that Billy begins to speak of an alien plant called Tralfamadore and time travel. Billy and the narrative both allege his ‘spasticity’ ‘began’ during combat, and that he travelled to Tralfamadore the year before the plane crash. Of course, the ‘spasticity’ makes a nonsense of any possibility of beginning, onceness or firstness. Presumably, according to the logic of the text, if he had met the Tralfamadoreans only in the last second of his life, it would have altered how he saw *all* of his life and there would have been no difference in the contours of the text of Billy’s (a-temporal) phenomenological experience. Regardless, the head trauma encourages the reader to follow the diagnostic thinking of Billy’s daughter Barbara: the lack of agency, flattened affect, delusional framework and disrupted experience of time have a neurological, organic aetiology (or set of causes). But the diagnoses proliferate. The famous ‘so it goes’ refrain in the face of death – recurring tens of times throughout *Slaughterhouse-Five* – could be pathologised as a dissociative, unempathetic symptom characteristic of impaired theory of mind (ToM), and a medical sign of autism, Asperger’s syndrome or ‘schizophrenia’ (Casacchia et al., 2004; Tanweer et al., 2010). The foregrounding of the term ‘schizophrenic’ and its explicit connection to the structuring of the text, of Billy’s phenomenological reality and of time-space itself, pushes the association between these temporal displacements and ‘psychosis’. By sad coincidence, Vonnegut’s eldest son Mark would receive a diagnosis of ‘schizophrenia’ in the years immediately following the publication of *Slaughterhouse-Five*, and he describes some of the temporal expansions, contractions and ellipses he experienced in his pathography^4^ *The Eden Express*:

Sometimes when I got back from my little cosmic jaunts it looked like no time at all had passed in my absence, but so much had happened to me that I felt I must have managed to cram a year or more into an instant of everyone else’s time. Other times when I came back it was as if I had been in some sort of suspended animation. Years had passed for everyone but me. One way or another I was out of step. That much was clear. (Vonnegut, 1975: 137)

These ‘cosmic jaunts’ out of time resonate with Billy’s ‘spasticity’. Deliberately, then, *Slaughterhouse-Five* invites comparisons between Tralfamadorean and ‘psychotic’ phenomenologies of time, or timelessness.

Clinical and research accounts of experiences such as Billy’s arguably have much to learn from him, and a consideration of deixis as the locus of what might be considered ‘pathological’ about these experiences. Formalised by Tulving (1985), autonoetic consciousness is the neuroscientific term for a hypothesised function of the mind underpinning the mechanism of episodic memory, whereby the subjectivity projects itself through time to a specific place and moment. As Tulving (2002: 3) asserts, the ‘where’ and the ‘when’ are essential elements in the formation of episodic memory. Predictably, a deficit model pervades the neuroscientific discourse on MTT and autobiographical memory in people with a diagnosis of ‘schizophrenia’ (Berna et al., 2016; Danion et al., 2007). Markowitsch and Staniloiu’s (2011) panegyric to episodic-autobiographical memory describes it as the ‘highest human ontogenetic achievement’ (p.16) before detailing how it is lacking or deficient in Asperger’s, autism, dementia and psychoses, implying a concomitant depletion of humanity there too. As a valuable counterpoint, Billy’s ‘spasticity’ in time demonstrates how a surplus of autonoesis can create the same observable characteristics, and what appears from the outside as ‘poor reality testing’ or emotional underinvestment and inappropriate affect, is actually a viable minoritarian phenomenology of time. Minoritarian, because, in the simple, literal sense, it is a phenomenology experienced only by a minority, at least at any one time. Also, minoritarian in the sense described by Deleuze and Guattari (2004) as a ‘subsystem or an outsystem’ (p.117) contrary to the constant, standard measure of the majority, and a position of creative, subversive assault against the power position of the majority. Viable, because who could not favour Billy’s serenity over the anxiety of the incarcerated or drugged ‘psychotic’, unable to have their account of temporality validated by others? Similarly, Metcalfe et al. (2012) argue that too little MTT decreases agency; for Billy too *much* ability to travel in time drains him of agency. Or, to put it differently, agency is an illusory symptom of the delusion of time.^5^

Time in *Slaughterhouse-Five* is alleged to not exist. Billy Pilgrim is abducted by aliens from the planet Tralfamadore and inducted into their a-temporal mode of perception. There is no ‘once’ within the text. In the ‘telegraphic, schizophrenic style’, the text comprises clusters of signs and symbols that might radically range over a human life span in a few lines.^6^ Entrances onto and exits from this dramatic scene are concertinaed. Edgar Derby – who is the first character to be mentioned, though not by name – is formally introduced and dispatched in a few lines:

One of the best bodies belonged to the oldest American by far, a high school teacher from Indianapolis. His name was Edgar Derby […] Derby was forty-four years old. […] That good body of his would be filled with holes by a firing squad in Dresden in sixty-eight days. So it goes. (Vonnegut, 1991: 60)

This treatment of a life narrative is characteristic of the text. It is not that time is excluded from the account. In fact, it is very rigidly present and foreground. But time is no longer a sequence of present moments; it is a set of loci. It is not a dynamic essence, but a static ornament; not a movement, but the coordinates. This can be clearly seen in the well-known line: ‘I, Billy Pilgrim […] will die, have died, and will always die on February thirteenth, 1976’ (p.103). In the Tralfamadorean mode of composition, stories are ordered according to these coordinates, and a static proximity is the compositional method. It is an a-chronological flight of association that excludes ‘passages’ of time, because time does not ‘pass’ in this method, but rather connects or conjoins geometrically. There is no ‘once’ to this way of experiencing the self, time or the world/here, as time is only a relative space between fixed loci. Indeed, according to an interview in 1972, Vonnegut had a clock that chimed the time twice, obliterating the ‘once’-ness of any moment (Allen, 1988: 58).

Consider the Tralfamadorean a-temporal perception:

All moments, past, present, and future, always have existed, always will exist. The Tralfamadorians can look at all the different moments just the way we can look at a stretch of the Rocky Mountains, for instance. (Vonnegut, 1991: 19)

Despite its a-temporality, Tralfamadorian reality retains a dispassionate linearity. The reader should suppose a somehow endless Rocky Mountains, of which any one life occupies a ‘stretch’. Of course, this re-establishes a form of ‘once’-ness. From some height, the stretch would be reduced to a point. It has linear unity and absolute formal boundaries. For all his ‘spasticity’ in time, Billy – unlike Connie – cannot travel beyond his ‘life time’. Without time, any point of departure is irrelevant. Again, the analogy is with a complete visual, geometric structure; a painting, rather than a narrative; a map, rather than a journey. As such, the story does not have a beginning, middle and end, but is composed of a centre and peripheries. In fact, the text is ordered around a missing – a traumatically repressed or, more accurately, excised – ‘centre’ as Vonnegut explained in an interview for *Playboy* in 1973:

The book was largely a found object. It was what was in my head, and I was able to get it out, but one of the characteristics about this object was that there was a complete blank where the bombing of Dresden took place, because I don’t remember. And I looked up several of my war buddies and they didn’t remember, either. They didn’t want to talk about it. There was a complete forgetting of what it was like. There were all kinds of information surrounding the event, but as far as my memory bank was concerned, the centre had been pulled right out of the story. (Allen, 1988: 94)

**Figure 3.**
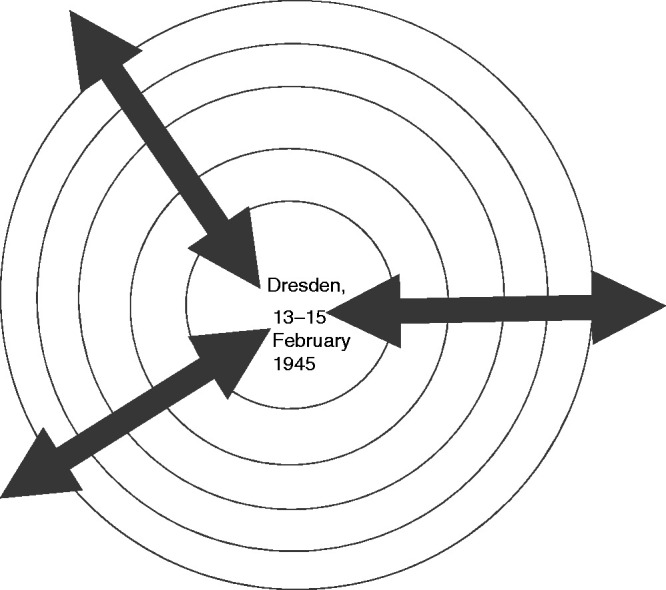
Schema of Billy Pilgrim’s life as a stretch of the Rocky Mountains.

A map of the static, traumatised temporal landscape of *Slaughterhouse-Five* orientated around a structuring absence can be constructed as in Figure 3, whereby the concentric circles represent what might otherwise be conceptualised as linear chains of causality, and the arrows represent the contracting constellations of the ‘telegraphic, schizophrenic manner’.

Billy re-institutes the temporal self as a bounded unity, except that a geometric linearity is substituted for a chronological one. Connie, conversely, is caught in a dynamically causal linearity with the future beyond her own life. The acts of her present disrupt and define the possibilities for the future. Just as the temporal relations are in flux, so is the identity of the narrative self within the tripartite relationship of deixis: self, here and now. The tug of creative, responsive, adaptive fluidity of the self against deadened fixity is nicely illustrated by Connie’s confusion at the ever-changing names of the inhabitants of Mattaposett: ‘You change your name any time you want to?’ (Piercy, 1979: 77) Self in Mattaposett – as signified by a name – is explicitly time-dependent, and breaks with former identities and selves are not the traumatic ruptures they are for a working-class woman of colour in 1970s America. In the same way, going ‘mad’ in Mattaposett is a deliberate process ‘to disintegrate, to reintegrate’ (Piercy, 1979: 66) rather than a carceral and pharmacological punishment, through which medical institutional power shatters the self diagnosed as ‘mad’. Later, Connie recognises that her immutable names – staging personal history as personal destiny in their unchanging implacability – represent a disintegrated or very literally schizoid self, with Connie, Consuelo and Conchita formalising and fixing different ethnic, class and social configurations of the self simultaneously (p.122). In this formulation, Connie embodies the same static, geometric temporality of Billy, although rather than being dispersed horizontally according to a ‘now’ – as Billy spasms across his stretch of Rocky Mountains – Connie is dispersed vertically according to a ‘here’. For some situations and people, she is Connie, for others Consuelo or Conchita. A crisis in the ‘here’ of her deictic relations provokes temporal displacement.

Consequently, the whole narrative of *Woman on the Edge of Time* can perhaps be reduced to one agent’s reclaiming of their temporal selfhood and agency. Connie’s movements in time restore her deictic relations, as she finds a ‘here’ and ‘now’ that recognises rather than assaults her mutable self, and this culminates in her performing a violent act of will that potentially alters the future Mattaposett. It certainly alters her immediate future and those of the research team using her as a guinea pig for a grotesque, invasive neurological ‘cure’ for ‘psychosis’. She regains her autonoetic orientation – previously stripped away through the systematic impoverishing, sexist oppression and psychiatric incarceration and torture she has endured – and thereby regains a future (albeit one that comes under threat and that she stands to lose, and is beyond the bounded unity of her own personal time and space). This model of the temporal self is one of radical futurism; those instances – bound to a person and a time – of self-agency have consequences that are, contrastingly, well beyond those bounds of person and time. They are collective and futural, dispersed in terms of person and time. Sam McBean casts *Woman on the Edge of Time* as staging a problematic of feminist futurity where ‘[t]he feminist present might be best imagined as persistently interrupted by the demands of the future – haunted by the promise of something different and something more’ (2014: 53). McBean captures the dialogic relationship between Connie’s present, which re-structured as past, serves to construct the future: ‘*Woman on the Edge of Time* not only functions as a critique of the present through the utopian genre, but also brings loss, mourning, haunting and futurity into close contact with each other – so that Connie’s past losses are formative and productive of the future’ (2014: 40). The restitution of Connie’s agency performs in microcosm the empowering human – and distinctively but not exclusively female – flourishing that Mattaposett potentiates (Trainor, 2005).

For Connie, time travel is an act of movement and presence. This is framed very specifically as a transportation to another ‘here’ and ‘now’ in which Connie has agency. When Luciente first suggests taking Connie to Mattaposett, she proposes taking a walk but ‘[n]ot a walk here or now’ (p.65). ‘Here’ and ‘now’ are so mutually dependent in these dislocations that they become synecdochally entwined. At the end of this first time trip for Connie, Luciente explains: ‘Understand, you are not really here’ (p.79). By here, Luciente also means now. An alternative dystopian future, or – and the commensurability is significant – an alternative social, environmental locus within the same future, opens up for Connie, potentially in response to the medical experiment she is undergoing in her own present time, placing her in the ‘168th and General File’ of New York (p.288). From this juncture, she experiences considerable difficulty in contacting Luciente and travelling to Mattaposett. The qualities of presence and movement, as constitutive of time travel, are now very evident:

Every day for a week she tried to *summon* Luciente but without success. Once she felt herself *slipping into* that other future, till she *drew back* with horror. Why couldn’t she *call* Luciente? Since they had implanted the dialytrode, she had not been able to *reach over* on her own, not the right future, the one she wanted.

[…]

[S]he cast herself on her bed and *flung herself* toward Luciente, she did not care how. The *going over was rocky*. For a time the ward dimmed and yet she did not arrive in the future. She passed out. It was more like fainting than falling asleep. But at last *she stood* with Luciente’s *hands on her shoulders* in a small clearing. (my emphasis, Piercy, 1979: 326–327)

Travelling to Mattaposett – ‘catching’ in Luciente’s terminology – is a physical act that requires mutual recognition, and a psychic metaphorical contact that literalises into a physical one. The benefits of such touch, contact and mutually recognising presence are obvious when placed in the context of Connie’s carceral environment in her own present.

Though many enduring mental distress and, more pointedly, mental health services in the 21st century will still find all too much that is familiar in Connie’s story, the importance of the ‘here’ of the deictic sign is increasingly recognised in health sciences research, giving sharper analytic tools for articulating woolly truisms about the worsening, maddening effects of social isolation, institutionalisation and incarceration. Specifically, co-presence and the role of shared ‘place’ for identity building and validation is a developing area of interest in social science-infused cognitive and neuroscientific research (Davis, 2016; Lengen and Kistemann, 2012; Lengen et al., 2019). For most of the novel, Mattaposett is a place of healing or at least respite for Connie. It is also one in which her deictic relations are restored. To recognise, to witness, to re-affirm the status of the deictic sign relationship is a practice of shared presence and presents, a community in the ‘here’ of contact as *Interpretant* and in the ‘now’ as mutually acknowledged *Object*.

## The Event and the structure of trauma

A Tralfamadorean has (and will, and always has and will) destroyed the universe and all existence: ‘He has *always* pressed it, and he always *will*. We *always* let him and we always *will* let him. The moment is *structured* that way’ (Vonnegut, 1991: 84). This, in their timeless phenomenology, is not a final moment, or a destination, but a structural textual effect. It cuts through, and returns in, every moment of the universe. The universe is, in fact, also the end of the universe. In much the same way as that the universe, given time relativity, was not *caused* by the big bang, so much as it *is* the big bang. There is only one moment and it is structured that way.

The analogous relationships between the bombing of Dresden and the structure of the text of *Slaughterhouse-Five* and the phenomenology of Billy’s ‘spasticity’, and his psyche, and that of the traumatised narrator, as textual feature, are very apparent. Vonnegut’s text performs a traumatic temporal dissociation so effectively that momentary interruptions by the author to reconnect him with his own lived experience – ‘I was there’ (1991: 49) ‘That was I. That was me.’ (1991: 91, 108) – are abrupt and absurdly comic. Their repetition and insistence recall the destruction of the universe, or Dresden, or the psyche as the eternally returning, structuring Event described by Gilles Deleuze in *Logic of Sense*:

[A]n eternal return which is no longer that of individuals, persons, and worlds, but only of pure events which the instant, displaced over the line, goes on dividing into already past and yet to come. Nothing other than the Event subsists, the Event alone, *Eventum tantum* for all contraries, which communicates with itself through its own distance and resonates across all of its disjuncts. (2004: 201)

The Event for Delueze is both timeless – as it is every-when, reverberating through time as a geometric, static construct akin to that in Figure 3 – and also a moment of pause or caesura: ‘the caesura, of whatever kind, must be determined in the image of a unique and tremendous event, an act which is adequate to time as a whole’ (Deleuze, 1994: 89). In the same way, it serves to both fracture and construct the self, and the temporality constitutive of and by that self.

Why is it useful to introduce the concept of the Deleuzean Event at this moment? As a true Deleuzean Event, ‘psychosis’ can resonate in any direction, without heed to causality, corporeality or temporality. The Event is the cut, or caesura, or explosion through which the self can be lost in time. And this occurs as observable autonoetic surplus (not deficit) in ‘psychosis’. The test for this kind of high theoretical thinking about ‘psychosis’ should surely be whether it is borne out in encounters with actual real evidence. Is empirical data supporting this model available in real-life examples? Since the late 1970s, *Schizophrenia Bulletin* has regularly published first-person accounts from people with a diagnosis of ‘schizophrenia’. Christina Bruni’s (2014) first-person account exhibits temporal distortions and ‘spasticity’ indicative of a deictic crisis. In a text of only a few hundred words, and which is presented as a tale of successful recovery, there are continual temporal signposts of specific dates and periods of time, but these again present a chronology that, despite its insistence, fails to hold together. Bruni attempts to nail each paragraph into a linear account, and yet the text flickers between moments that seem, at best, under-integrated:

Over 6 years […] a 9-year run […] Since 2002 […] In June 2000 […] over 12 years […] a 7-year gig […] In 1990 […] Friday, September 25, 1987, at five o’clock […] 9:00am that Saturday […] 24 h […] 3 weeks […] over 20 years […] April 1992 […] 2 weeks […] spring of 1998 […] Five years into this […] since July 2003, going on 10 years […] April 2007 […] a week before my birthday […] the next day […] that April night in 2007 […] For 16 years […] Within 3 days […] the fall of 2007 […] in 1987 […]. (Bruni, 2014: 3–4)^7^

Preoccupied with isolating and delineating her ‘breakdowns’ as ‘episodes’ hermetically sealed off from the on-going life narrative of recovery and remission, the text reads like the anti-chronology of the Deleuzean Event.

Similar representations of the Deleuzean Event can be seen through an analysis of research interviews with people living with a diagnosis, conducted by researchers at the University of Birmingham contemporaneous with Bruni’s account.^8^ The trauma *of* psychosis (which may or may not be the aetiological trauma *prior* to psychosis) violates the fixity of deictic relations constitutive of communication, and the medical, temporal and social semiotics. When an interviewee recounts an instance of voice-hearing, consistency of tense is lost as the ever-present Event is re-staged in the telling:

I was, for this one I was in the bathroom, that one I was in my room, the first one I was in my room and the second time I was in the bathroom and then the third time I was in the front room, so wherever I am in the house they started. Like they’re starting on me now that I’m remembering them, just doing the entry. I went to have a relaxing sit down in the front room and the voices are saying “Get up, get up”.

Psychiatric and neuroscientific research into the lived experience of time in ‘psychosis’ already offers theories for understanding what is taking place here, so what advantages or contributions does this Deleuzean analysis potentially bring? Take for example Vogeley and Kupke (2007), a paper by a psychiatrist and a philosopher respectively, which deploys Husserl’s phenomenological model of the tripartite orientation in time: ‘retention (as the intentional act directed to the past), protention (as the intentional act directed to the future), and presentation (as the intentional act directed to the present)’ (p.158). It is this orientation that appears disturbed for this interviewee. Yet by retaining the structuring concept of the Deleuzean Event, the previously pathologised speech can be re-evaluated as a rich source of phenomenological evidence.

Rather than being instrumentalised as a sign of neuroanatomical malfunction, and incorporated into a short-circuiting semiotic exchange linking a vague diagnostic term to an inaccessible and enigmatic subjectivity, the temporal disturbances, the ‘spasticities’ in time, speak of an existential truth for an embodied, historically and socially situated individual suffering (or perhaps not suffering and only experiencing). And this speech act, interpellating any complicit listeners into a deictic arrangement that witnesses and confirms the effaced subjectivity of the speaking voice, forces a sharing of the subjective experience of voice-hearing. This is an unavoidable semiotic effect. The ‘here and now’ of the utterance brings, as a grammatical, deictic, textual consequence, the ‘here and now’ of the hearing of the voices described, collapsing these two points in time in the style of the geometric temporality of Tralfamadore: ‘wherever I *am* […] they *started* … I *went to have* a relaxing sit down […] and the voices *are* saying […].’ There is no way to not enact and to not hear the voices when speaking of them, for a voice is never more nor less than a voice. To remember the voice is to re-hear the voice, and to re-hear the voice is for the voice to be speaking, for speaking is only ever the moment of speaking. The voice-hearer, regardless of the details of how and in what way they hear voices, is always in the Event of voice-hearing, when they hear them, and when they remember or speak of hearing them.

The voices appear *first* and *then* acquire pronouns. They take them as a linguistic, semiotic and deictic necessity. Observe how they progress from a focalised, depersonalised (or pre-personalised) state of pure voice – ‘the voices were going to make you drop it’; referring to themselves as ‘you’ the interviewee adopts the voices’ deictic position, but leaves it unvoiced – through a fusion with the interviewee’s first person singular position (‘I’m going to push’) until they reach a full realisation in the first person plural of their complete characterisation:

Then I was making some honey water for myself and the voices were going to make you drop it, so sometimes if I’m carrying something like a drink, a drink of water or some water, my thoughts will start off like ‘It’s going to drop’ and, it’ll get like, if I’m holding it like, the thought will be like ‘Oh it’s going to drop, I’m going to push your arm’ or something like that. […] Like, they’re not going to do it but my arm was just going to move. The voices ‘We’re going to make you drop it’ and the voices ‘We’re going to get you’[.]

That this linguistic procedure, in the uttering, mirrors the voices’ increasing agency, presence and influence over the interviewee, in the event being uttered, is no coincidence; this is because these are *one and the same thing*.

This is not to say that psychosis is the hearing of voices. In fact, the argument is more to the contrary: the hearing of voices can be understood as a language effect of a psycho-social trauma or Event. The hearing of voices is, in some cases, clearly secondary. To an extent, voices may only come into (full) being when recounted for another listener. In this, voices operate like (Peircean) signs: they require the third figure of the *Interpretant*, as described at the start of this paper. The voices, then, are a sign created in the semiotic act. This stands whether the *Interpretant* position is occupied by an external other – the interviewer – or is an internal figure in a mentally voiced semiotic exchange. The tense shifts are not a mistake or agrammatism. They are the Event becoming immanent in language. This is the process Deleuze and Guattari would later term counter-effectuation (1994: 159). However, when the Event’s representation in language is inseparable, or non-representational, in relation to its abstract pure existence, then the counter-effectuation undoes itself. This is the potentially unique quality of the temporality of voice-hearing specifically and ‘psychotic’ time travel generally.

## Warnings; explanations

‘All time is all time. It does not change. It does not lend itself to warnings or explanations. It simply *is.*’ (Vonnegut, 1991: 62)

There are directly therapeutic benefits to be gained from this analysis, perhaps. Sonja Bar-Am (2015) writes of the potential of using magical realist genre texts and a Deleuzean rhizomatic approach to the construction of the therapeutic space and relationship. Mostly, Bar-Am’s paper details her successes using magical realism texts, and specifically their characteristic blending of folkloric, mythic and dreamlike material with an essential realist framework, to open up discussions with people with lived experience of psychosis without shutting down their own magical realist accounts as pathological, delusional or psychotic. It is hoped that this analysis, using two very different SF texts, has demonstrated that other genres beyond magical realism have something to offer, that these texts have a content of form, as well as a content of content, and that the implications and insights apply not just to therapeutic and clinical situations but also to theorising about the structure of psychosis.

Certainly, *Woman on the Edge of Time* has some simple therapeutic lessons. Stories, and specifically stories of feminist alternatives, are the only successful remedy available to Connie and her fellow ‘in-patients’ for the hollowing time-space of incarceration. They reclaim and reinvigorate the time-space, and the selves within that: ‘[h]er telling took them through the supper lines and the supper of what Sybil pronounced Toad Stew, through the evening medication line and the blank space of time until lights out’ (p.84). Similarly, Connie’s murder of the clinical researchers experimenting on her, in order to save the future Mattaposett, can be read as a pre-emptive riposte to research papers such as Raffard et al. (2016), who suggest that ‘imagining a positive future is important in developing an optimism bias’ (p.140) in countering temporal displacements and psychosis. Connie does not choose to imagine a positive future, although that might – in a literalist reading – be what she is doing when time travelling. Instead, she fights in the present for a better future. *Woman on the Edge of Time* is a story about taking back one’s place (and specifically positing a ‘one’ that transcends carceral gender and racial determinations). There is some considerable contrast with *Slaughterhouse-Five*’s ‘so it goes’. Billy’s ‘spasticity’ drains any present moment of its agency (and its agent: him). Connie’s ‘catching’ empowers her, and makes her present moment pivotal. Billy’s present self is lost in time, whereas time is found within Connie’s present self. This contrast establishes some parameters for calibrating autonoesis. It is implausible for the self to be within their experience, within the very experience through which the autonoetic self is constructed. To be stuck within the carceral present, for Connie, would mean losing her future (and the future beyond her) and so destroying her agentive self. Conversely, going too far or too ‘spastic’ in time, detaches the self from any ‘here and now’. At either extreme, being without time is being without self.

In effect, this analysis of the Vonnegut and Piercy’s texts, the first-person accounts and interviews shows what specific case studies of individual instances of temporal ‘spasms’ can tell us about the relations between individual’s social and temporal orientations. But to have any serious explanatory or predictive power, the Deleuzean model needs more detail. This paper proposes a way of amalgamating the geometric structure of time engineered from these primary texts (Figure 3) with Deleuze’s Event, which brings along its own geometric imperatives. The key to this unification is the chief problematic of time, perhaps even the chief problematic of existence: how is an infinitude of time accommodated within a subjectivity that is necessarily temporally finite? How can the unbounded time, time *as* time, and bounded time, time *as* subjectivity, operate within the same system, be it the psychic experiential phenomenological system of the individual mind, or a conceptual model for theorising about time? Fascinatingly, the answer might lie in precisely that element pathologised as ‘schizophrenic’ or ‘psychotic’ – the caesura that constitutes the ‘psychotic break’ and the point of ‘psychotic’ temporal displacements.

Deleuze models this caesura or rupture on the Dedekind cut (1994: 172), named after the mathematician Richard Dedekind who proposed a perfect, linear set of real numbers (both rational and irrational). The mathematics only needs to be understood metaphorically here. Consider an irrational number as cutting through a sequence. Irrational numbers – such as π – are boundless and non-repeating; a clear analogy for a certain mode of time that is depersonalised and eternal. Deleuze’s third synthesis of time outlined in *Difference and Repetition*^9^ allows for the incorporation of this infinitude within a geometric static temporal model, whereby an irrational number constitutes a vertical infinitude as caesura in a linear and – in the context of a temporal subject or Tralfamadorian stretch – finite range. It corresponds to the Aion, as described by Brighenti and Kärrholm in this journal: ‘Aion is the endless infinitive line of the instant. The Event, therefore, ranges across infinite time, whereas the life is a static time’ (2019: 378). As a theoretical construct, this allows for the unification of boundless moments, and eternally returning events (and selves) within a still bounded and static life.^10^

However, Deleuze’s model is not cold abstraction. It is an ethical recognition of lived reality for the marginalised and traumatised. As Marxist linguistic Jean-Jacques Lecercle asserts, it calls for a re-affirmation of the real existence of embodied selves and the real relations between them:

The philosophical line of separation, therefore, runs between events and things. Hence the paradoxical nature of the event: it is the result of the mixture of bodies, it is produced by such mixture, but it is of a different order, outside the bodies, outside their actions and passions, outside the time in which they unfold. (Lecercle, 2002: 114)

**Figure 4.**
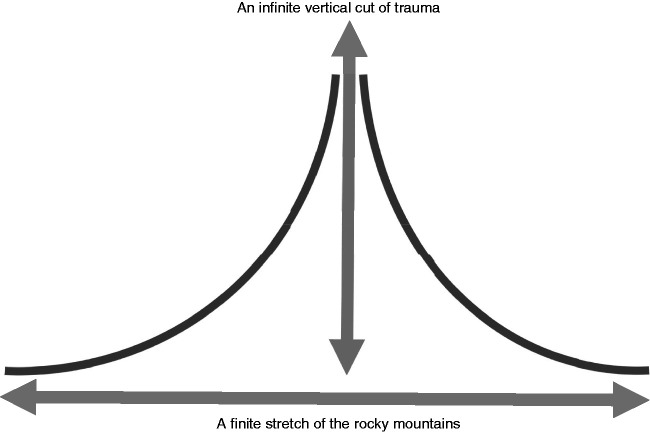
A model of traumatised, ‘psychotic’ time. The thick grey line without arrow heads represents the self experiencing time-space in deictic relations. The space in the rupture — where the infinite vertical cut of trauma occurs — is ‘psychotic’ time, not experienced or experienced according to deictic crisis.

This Deleuzean synthesis of time offers a model that is neither the narrative nor the episodic model of structuring a self that endures through time, and this model can be represented by Figure 4. It is a model that not only accommodates but is actively reliant upon incomprehensible, unmappable cuts that simultaneously order and disrupt the subject’s synthesis of time. Within the cut, spiralling to infinity, the self neither experiences itself, nor time-space (‘here and now’). These cuts are both made by deictic crisis and offer a structure of response to it. The value of the Deleuzean model when applied to people with a lived experience of psychosis is that rather than discarding their phenomenological experience of time as a pathological symptom, it is able to hold these experiences within an explanatory framework with other non-psychotic experiences. The psychotic experience is not aberrant and its difference might be seen as analogous to the difference between rational and irrational numbers within a perfect set of real numbers.

Psychosis then is a time bomb of sorts. Not a bomb that only goes off at a designated time, but which also contains an infinite vertical stream of time inside itself that creates a rupture within the linear unity of the life. Connie rides the time-blast to be flung into a distant future, where she is re-stabilised (for a time) through deictic validation. Billy takes this energy and explores and comes to terms with the traumatic contours of his life. In both examples, the temporal rupture is not only a process of disintegration but also, and simultaneously, of reintegration, as with the ‘madness’ process in Mattaposett. So, what sort of self is reconstituted through this model of time?

As for the third time in which the future appears, this signifies that the event and the act possess a secret coherence which excludes that of the self; that they turn back against the self which has become their equal and smash it to pieces, as though the bearer of the new world were carried away and dispersed by the shock of the multiplicity to which it gives birth: what the self has become equal to is the unequal in itself. In this manner, the I which is fractured according to the order of time and the Self which is divided according to the temporal series correspond and find a common descendant in the man without name, without family, without qualities, without self or I, the ‘plebeian’ guardian of a secret, the already-Overman whose scattered members gravitate around the sublime image. (Deleuze, 1994: 89–90)

Nietzschean ‘Overman’ is not the most immediately obvious model for Billy Pilgrim or the person in a crisis of deixis. Perhaps there is more of a resemblance with Connie and her retributive, redemptive violent act of re-establishment in the deictic order. But this model of the traumatised temporal structure of psychosis allows for conceptualising the phenomenological experience of the person with lived experience of psychosis, for (imperfectly perhaps) re-integrating them into deictic arrangements without over-writing or ignoring their accounts of their reality, and for attending to and witnessing their self-validated meanings. It is a matter of recognising the temporalities they bring and construct, and hearing their ‘brief urgent message’. This is not an act of pitying empathy merely; as Connie demonstrates, the urgency may not be for the minoritarian (the female, the person of colour, the ‘psychotic’); it may be an urgent message for the majoritarian structure whose moment of explosion is at hand.

## Notes

1 *Diegesis* (meaning ‘narration’) originates as a term in contradistinction to *mimesis* (‘imitation’) in classical Greek explorations and typologies of poetry, notably Plato’s *Republic* and Aristotle’s *Poetics*. Here, the usage is drawn from current narratological and semiotic discourses, and as used in this paper is well-defined by Gérard Genette as pertaining to ‘the universe of the … narrative’ (1980: 228). Anything diegetic is that which takes place within the universe of the narrative.

2 The expression ‘telegraphic schizophrenic manner’ comes from the unpaginated frontispiece to *Slaughterhouse-Five*.

3 It is worth clarifying – to avoid unintended offence – that in Vonnegut’s phrase, ‘spastic’ means ‘of the nature of a spasm or sudden involuntary contraction’ rather than being any reference to cerebral palsy, let alone a derogatory one (*New Shorter Oxford English Dictionary*, 1993: 2968). While there is now due sensitivity over the use of a word like ‘spastic’, even figuratively, much less commonly are such serious reservations held or emotional affordances made for the word ‘schizophrenic’. Both will be used but in scare quotations to indicate awareness of how problematic they are as terms.

4 Pathography, illness narratives or memoirs are commonly first-person narratives of a real lived experience of a disease, and constitute the pre-eminent object of interest for medical humanities literary scholarship. The term pathography was popularised in the last decade of the 20th century, by theorists of illness writing such as Thomas Couser, Arthur Frank and Kathryn Montgomery Hunter, although pathographies as texts obviously pre-exist their critical formulation as a genre. For accounts of the early history of pathography criticism, see Hawkins (1999a, 1999b).

5 The bias is arguably disciplinary. Where cognitive neuroscience sees a deficit, psychology sees an increased capacity, as in Winfield and Kamboj (2010) which correlates greater sensitivity to autonoetic awareness with positive schizotypy.

6 Vonnegut had previously formulated the Tralfamadorean temporal perspective without reference to ‘schizophrenia’ as ‘chrono-synclastic infundibula’ in *The Sirens of Titan* (1959). See Rubens (1979) for an account of Vonnegut’s refinement of his model of time.

7 Even the title of the account, ‘Immediate intervention: life-long success’, speaks to a temporal *Spaltung*: the ‘immediate’ and the ‘life-long’ pull in opposite directions.

8 These transcripts were supplied by and are used with the permission of Dr Rachel Upthegrove, Institute of Clinical Sciences, University of Birmingham. This section analysing the transcripts and Bruni’s first-person account of living with a diagnosis of schizophrenia is drawn from the author’s unpublished PhD thesis.

9 Originally published in French in 1968, it coincides not only with the May revolution but also with Billy Pilgrim’s plane accident.

10 For a brief account of Bertrand Russell’s and Wittgenstein’s differing critiques of Dedekind’s spatial analogy of the cut, and the problem with irrational numbers bisecting a continuous, complete number system, see Voss (2013: 202–204).
